# Medical Association of Georgia

**Published:** 1875-03

**Authors:** 


*Medical Association of Georgia.*

This body will hold its annual meeting in Savannah, Ga., April 21st inst. We trust the profession of the State will not let the opportunity slip to attend the deliberations of this Association, where professional profit may be had and old friendships renewed. Owing to circumstances beyond our control, the editors of the RECORD cannot share this pleasure with them, but trust that the meeting will be well attended, and the best interests of medicine subserved.

